# Difficulty interpreting the results of some trials: the case of therapeutic hypothermia after pediatric cardiac arrest

**DOI:** 10.1186/s13054-015-1121-4

**Published:** 2015-11-09

**Authors:** Jean-Louis Vincent, Fabio S. Taccone

**Affiliations:** Department of Intensive Care, Erasme Hospital, Université Libre de Bruxelles, Brussels, 1070 Belgium

Therapeutic hypothermia or targeted temperature management (TTM) is widely used after cardiac arrest (CA) in adult patients and improves survival and neurologic outcome after out-of-hospital CA due to shockable rhythms [1]. However, several issues, including the optimal target temperature, the time to initiate cooling and the most effective duration, remain controversial in this setting [2]. In neonatal hypoxic encephalopathy, randomized controlled trials (RCTs) have shown that cooling comatose newborn patients for 72 hours, especially when brain damage has been demonstrated by altered electroencephalographic findings, improves the proportion of patients with an intact neurological recovery [3, 4]. The results of clinical trials in the setting of pediatric CA are, however, difficult to interpret because RCTs are lacking. A recent meta-analysis of six studies (three retrospective and three prospective cohort studies, total n = 356) showed that the evidence supporting TTM in this setting was poor [5].

When we read the conclusions of the recent RCT by Moler et al. [6], which investigated two early TTM strategies (33.0 °C versus 36.8 °C) in 295 comatose children after out-of-hospital CA, we initially considered it as a negative trial providing more evidence against the brain-protective effects of post-CA hypothermia in this subset of patients. But when we read the paper more carefully, we realized that perhaps a different conclusion could be reached. Among the 260 children with available data on outcome, 20 % of those cooled to 33.0 °C and 12 % of those managed at 36.8 °C had an intact neurological recovery (*p* = 0.14). The *p* value is indeed 'non-significant', but in real life, statistics may not tell the full story. Although the analysis was conducted according to our current methodological standards using rigorous statistical evaluation, statistics are not always the only relevant factor in real-life individual patient management; clinical judgment, assessing the likely risks and benefits of each proposed strategy, also plays a role.

To try and highlight the clinical limitations of statistical reliance when interpreting study results, imagine the following conversation the next time you talk to parents of a child who has had a CA:

*Doctor:* I’m really sorry, but your child may have serious brain damage as a result of his cardiac arrest.

*Parent:* That’s terrible! Isn’t there anything we can do?

*Doctor:* I’m afraid not. There are some interventions that have been suggested, but they’ve not been shown to be effective.

*Parent*: What interventions?

*Doctor:* Well, cooling the body for a couple of days, for example. It’s been tried in neonates with birth asphyxia and adults after cardiac arrest.

*Parent*: But … if this intervention is used in neonates and adults, how can you say it won’t work in children?

*Doctor*: Well, in a recent study including almost 300 children, 20 % of those who were cooled survived with good brain function versus just 12 % of those who weren’t cooled. Neurological status improved in 38 % of the cooled children compared with only 29 % of the non-cooled. And, 28 days after the arrest, the mortality rate was 10 % lower in cooled children (57 % versus 67 %). Unfortunately, when the researchers applied the standard statistical rules that we use to interpret all scientific research, there was more than a 10 % possibility that these differences were due to chance, so we can’t recommend it.

*Parent:* But those results are really encouraging. Even if statistics tell you that this may be due to chance, there’s still the possibility that it wasn’t and I’d like my child to have that opportunity. Maybe the treatment’s expensive?

*Doctor:* No, that’s not the issue.

*Parent:* Was it dangerous then?

*Doctor:* Quite safe actually. Potassium and platelet levels went down a little, but with no harmful consequences. There is a risk that the heart rhythm can be affected; some of these abnormalities can even be quite dangerous. In the same study, serious abnormalities of the heart rhythm occurred in 11 % of the cooled children and 9 % of the others. Reduction in body temperature also increases the risk of infections; the investigators of this study reported that 46 % of cooled children developed an infection, compared with 39 % of the other children.

*Parent*: So, the treatment is associated with some risk but can still improve the chances of my child surviving… how can you balance the benefits and the risks for my boy?

*Doctor:* Honestly, I don’t know. If I just have to use numbers… 12 children would need to be cooled instead of kept at normal temperature in order to have one additional child with a good clinical outcome. And, 15 children would need to be cooled for one child to develop an infection.

*Parent:* Please, try this treatment on my child.

## Abbreviations

CA: Cardiac arrest
RCT: Randomized controlled trial
TTM: Targeted temperature management
